# Emotion Regulation Strategies Can Predict Task-Switching Abilities in Euthymic Bipolar Patients

**DOI:** 10.3389/fnhum.2014.00847

**Published:** 2014-10-27

**Authors:** Amara Gul, Kamran Khan

**Affiliations:** ^1^Department of Applied Psychology, The Islamia University of Bahawalpur, Bahawalpur, Pakistan

**Keywords:** executive function, cognition, emotion, face categorization, cognitive reappraisal, emotion suppression

## Abstract

This study examined task-switching abilities and emotion regulation strategies in euthymic bipolar patients (EBP). Forty EBP and 40 healthy individuals performed face categorization tasks where they switched between emotion and non-emotion (i.e., gender) features among faces and completed emotion regulation questionnaire (Gross and John, 2003). Subject groups showed substantial differences in task-switching abilities and emotion regulation strategies: (1) there was a dissociation between emotion and gender classification in EBP. The switch cost was larger [i.e., higher reaction times (RTs) on switch as compared to no-switch trials] for gender categorization as compared to the emotion categorization task. In contrast, such asymmetries were absent among healthy participants. The differential pattern of task switching reflected functional disturbances in frontotemporal neural system and an attentional bias to emotion features of the faces in EBP. This suggests that when a euthymic bipolar patient is preoccupied with emotion recognition, an instruction to perform gender categorization results in greater cost on RTs. (2) In contrast to healthy individuals, EBP reported more frequent use of emotion suppression and lesser use of cognitive reappraisal as emotion regulation strategy. (3) Emotion regulation was found to be a significant predictor of task-switching abilities. It is argued that task switching deficits rely on maladaptive emotion regulation strategies in EBP specifically when tasks of emotional significance are involved.

## Introduction

Bipolar disorder (BD) is an abnormal mental state that is characterized by periods of elevated mood and depression (Anderson et al., 2012). The elevated mood is categorized as mania or hypomania depending on the severity of symptoms. Mania is associated with abnormal happy, overly excited, or extreme irritable mood. Manic individuals make decisions without anticipation of consequences. Depressive periods are accompanied by intense sad mood, poor eye contact, and negative schema of the world (American Psychiatric Association, 2013). Euthymia in BD is a state of stable and reasonable positive mood. The mood stability in euthymic bipolar patients (EBP) refers to the absence of manic or depressive periods. Cognitive and emotion function is disturbed across manic, depressive, and euthymic states in BD (Murphy et al., 1999, 2001; Martínez-Arán et al., 2004; Phillips et al., 2008), However, it is unclear whether higher order cognitive functioning such as task switching and emotion processing is disturbed in euthymic states. We therefore examined task-switching abilities in EBP. Furthermore, we investigated the effect of emotion regulation strategies on task-switching abilities in EBP.

### Task switching

Task switching is an executive function that is mediated by regions of the prefrontal cortex (PFC): dlPFC is involved in response inhibition/set-shifting, anterior cingulate cortex mediates decision-making (Alvarez and Emory, 2006), and orbitofrontal cortex controls emotional experience (Lezak, 2004). Switching is a kind of cognitive flexibility to shift attention from one task to another that enables an individual to rapidly shift and adapt to new situations (Monchi et al., 2004).Task switching deficits have been observed in patients with prefrontal dysfunction (Sawada et al., 2012).

In task-switching experiments, participants are instructed to perform two tasks in an alternating sequence. The response is made according to the changed task-set rule, therefore a switch cost may arise due to the subsequent delay in the selection of changed task-set rule/relevant stimulus-response-set (Rogers and Monsell, 1995; Mayr and Kliegl, 2000). Task switching requires two important mechanisms: (i) simple activation of the relevant rule and (ii) extra inhibitory processes to lessen interference from the competing task-set (Mayr and Kliegl, 2000; Rubinstein et al., 2001). These mechanisms are dissociable in the PFC. The left PFC controls the selection/activation of task-sets whereas the right PFC mediates the process of task-set inhibition (Brass and von Cramon, 2002; Aron et al., 2004). Human adaptation to new situations depends on the coordination of attention and cognition that can be experimentally evaluated by task-switching paradigm (Jersild, 1927; Rogers and Monsell, 1995). The task-switching paradigm examines control processes that require configuration of mental resources/state (e.g., attention, memory) when a task switches. Mental resources or states must be in accordance with the particular operations demanded by the task. Theory of task set reconfiguration states that once a task is switched, it remains activated until the new task-set is implemented and adapted. Switch costs arise from executive control processes that modulate reconfiguration of the cognitive system to implement a new task-set (Schneider and Logan, 2005). The idea of task-set inhibition asserts that switching between tasks requires suppression of the just completed task to adapt a new task-set (Mayr and Kliegl, 2000).

### Task switching and mood states

The challenging environment simultaneously involves two cognitive functions: first, maintenance of intensions and goals and second, switching between thoughts, actions, and goals according to environmental demands (Goschke, 2003; Dreisbach and Goschke, 2004). Mild positive effect reduces maintenance capability in a simple cuing paradigm (AX continuous performance task) and results in costs when a “to be maintained goal” is performed. In contrast, a benefit arises during sudden shift of the “to be maintained goal” (Dreisbach and Goschke, 2004). Behavioral studies suggest that positive effect increases cognitive flexibility (Isen and Daubman, 1984; Isen et al., 1987, 1992; Greene and Noice, 1988). Neuroimaging studies suggest an interaction between higher order cognition and affect (for review Dalgleish, 2004) that is mediated by dorsal and ventral regions of the PFC (Yamasaki et al., 2002). Savine and Braver (2010) demonstrated that performance improves on incentive trials, which results in low switch costs. This mechanism is associated with activations of reward centers of the brain (i.e., dopaminergic midbrain, paracingulate cortex).

The relationship between task switching and mood has been shown in studies of individuals with mood disorders. For example depressed individuals demonstrated task switching deficits due to focused attention on distressed stimuli (Whitmer and Gotlib, 2012). On the other hand, positive mood states also impair switching performance (Phillips et al., 2002) because positive mood may lead to amplified activation of task irrelevant information in working memory. This in turn leads to greater flexibility and originality sacrificing inhibition for irrelevant information (Dreisbach and Goschke, 2004; Goeleven et al., 2007; Rowe et al., 2007). This relationship remains significant even after controlling working memory, reaction time (RT), and inhibitory processes (Chu, 2014). Emotional engagement impairs the retrieval of mood incongruent memories (Greenberg and Meiran, 2014). Positive mood asserts a top-down adaptive process that is enforced on one’s internal structure to respond to situations. On contrary, negative mood imposes a bottom-up process, where internal structures are modified due to having external pressures (Fiedler, 2001). Motivation for the present study was to examine task switching in bipolar euthymic states.

### Task switching and emotion regulation

Intact emotion regulation abilities are associated with normal functioning of the frontal lobe whereas deficits in emotion regulation abilities are linked with abnormalities of the frontal lobe (Royall et al., 2002; Stuss and Levine, 2002; Gazzaley and D’Esposito, 2007). Executive functioning is positively related with downregulation and upregulation of emotions (Gyurak et al., 2012) and functionally engages common regions of the frontal lobe (Ochsner et al., 2002, 2004). The evidence of the relationship between executive functions and emotion regulation comes from various aspects of the research in cognitive science. A core finding is that people with high cognitive executive functioning demonstrate efficient emotion regulation abilities (e.g., Schmeichel et al., 2008). Better cognitive functioning reflects high inhibitory control that is instrumental in suppression of socially inappropriate expressions (von Hippel and Gonsalkorale, 2005). Subsequent studies found that efficient cognitive functioning is related with success in emotion suppression and cognitive reappraisal (Schmeichel et al., 2008; Schmeichel and Demaree, 2010). McRae et al. (2012) examined the relationship between several different cognitive abilities including working memory capacity, set-shifting ability, verbal ability, abstract reasoning, inhibitory control, and cognitive reappraisal. McRae et al. found that success at reappraisal was associated with working memory capacity and set-shifting ability. It has been noticed that people with high cognitive abilities have few mood changes when they suffer daily hassles (Stawski et al., 2010). The evidence is still scarce pertaining to task switching.

The ability to switch attention from emotions is associated with emotion-regulation abilities (Jhonson, 2009). Emotion regulation is the cognitive control of emotions that is operated on psychological, behavioral, and experiential level. On psychological level, individuals may change the thinking pattern associated with the event to feel positive. On behavioral level, individuals may avoid exposure of events to escape from negative feelings. Experientially, individuals may choose aspects of the events to attend (Gross and Munoz, 2006). Learning more effective emotion regulation strategies is useful in reducing psychopathological behavior (Hollon et al., 1992). Reappraisal reduces amygdala activation, thus an intensity of the emotional experience and physiological response is decreased (Phillips et al., 2008). People have tendency to behave in a certain manner, which becomes prioritized when the other task is not paid attention. The automaticity can be overridden by paying attention to the other task at hand (Peake et al., 2002). Reappraisal is the cognitive strategy that creates a balance between prioritized task and the other task that is in competition (Magen and Gross, 2007).

Emotions can also be modulated through suppression that leads to inhibition of ongoing emotional behavior (Gross and Levenson, 1993). Expressive suppression has adverse effects on health for instance an increased sympathetic nervous system arousal (Harris, 2001). Expressive suppression leads to impaired memory and social communication (Butler et al., 2003; Richards et al., 2003). Expressive suppression decreases attentional resources for encoding and enhances the recall of external events (Ellis and Ashbrook, 1988). Successful emotion suppression engages individuals in internal dialog where individuals remind themselves to suppress unwanted emotional impulses. This cognitive effort occupies attentional resources, as a result other cognitive activities might suffer (Pyszczynski and Greenberg, 1987).

Research on emotion regulation in BD has shown that patients with BD show increased magnitude on eye blink following removal of positive photographs and sustained elevations in positive emotions across various emotional contexts (Forbes et al., 2005; Gruber et al., 2008). Patients with BD show deficits in downregulation of emotions and tend to dwell on positive feelings following positive life event (Johnson et al., 2008).

### The present study

The present study was designed to examine the following objectives: (i) to compare switching abilities between emotion and non-emotion task (i.e., gender) in EBP versus healthy individuals and (ii) to assess whether task-switch costs are related to emotion regulation strategies in EBP and healthy individuals. We tested the hypothesis that EBP would exhibit greater switch cost for gender (non-emotion task) than the emotion task. Task switching requires response inhibition to currently irrelevant stimulus dimension (Thompson et al., 2005; Pattanayak et al., 2012; Camelo et al., 2013), and euthymic states are associated with disinhibition (Kaladjian et al., 2009) and decreased dlPFC activity in response to emotions (Hassel et al., 2008; Foland-Ross et al., 2012), we expected that EBP would show sustained attention for facial emotion. Second, we hypothesized that EBP would report higher maladaptive strategies (i.e., higher emotion suppression but lower cognitive reappraisal) compared to healthy individuals. This hypothesis was based on previous finding that BD individuals have tendency to indulge in maladaptive strategies to regulate emotions (Feldman et al., 2008). Third, emotion regulation strategies would predict task switch costs. This hypothesis was based on the finding that emotion regulation abilities that pertain to the engagement of goal directed behavior are compromised in EBP (Becerra et al., 2013).

## Materials and Methods

### Sample

Forty patients (18–35 years; *M* = 32.44, SD = 1.99) with bipolar I disorder currently euthymic were diagnosed by structured clinical interview for DSM-IV at Bahawal Victoria Hospital. Patients with bipolar I did not met the criteria for current manic, hypomanic, or depressive episode according to DSM-IV. Euthymic state was confirmed by the score of ≤7 on the Young mania rating scale (Young et al., 1978) and Hamilton depression rating scale (Hamilton, 1960). Patients were un-medicated at the time of diagnosis and testing. Half of them were female. Patients had average intellectual functioning as assessed by standard progressive matrices (Ravens, 1938).Characteristics of the sample are presented in Table 1.

**Table 1:** **Demographic and clinical characteristics for EBP and healthy controls**.

Variables	EBP (*n* = 40)	Controls (*n* = 40)
	*M* (SD) (min–mix)	*M* (SD) (min–mix)
Age at the time of testing	32.44 (1.99) (18.00–35.00)	28.48 (1.80) (18.00–35.00)
Age at euthymic onset (years)	31.00 (0.86)	
Disease duration in years	1.4 (0.55)	
Frequency of euthymic states per 4 weeks	20.00 (0.75)	
HAM-D	6.00 (0.57)	–
YMRS	5.50 (0.60)	–
Gender
Female	20	20
Male	20	20
Economic status
Lower	05	03
Middle	18	17
Higher	17	20
Education
Primary	–	–
Secondary	15	15
Higher	25	25
Intellectual function	Average	Average

Forty healthy subjects (18–35 years; *M* = 28.48, SD = 1.8) matched on age, gender, and intellectual function with no personal/family history of mental illness were contacted by advertisement from the local community. Participants had no psychotic and neurological symptoms, hypertension, history of head trauma, close relatives with psychiatric, and substance abuse disorder.

### Instruments

#### Task switching

##### Experimental stimuli

Forty-eight colored photographs were used as stimuli in the task-switching experiment. Half of the photographs were male faces (i.e., 24 photographs) and other half were female faces (i.e., 24 photographs). Photographs portrayed neutral and happy expressions of emotions. Photographs were standardized on bitmap image size of 288 × 288 pixels, bit depth 24 in white background. In the pilot study, participants were asked to rate the expression of facial emotion in photographs: “How would you describe salience of happy emotion on the scale from 1 to 10 (1 = low salience, 10 = high salience).” Photographs were selected on mean (standard deviation) ratings as salience [happy 8.04 (0.51) neutral 8.50 (0.65)] from a pool of 60 photographs, based on the ratings of a sample composed of 40 participants not participating in the present study (20 EBP and 20 healthy individuals) with an inter-rater reliability of 0.76.

##### Computer tasks and display

The experiment required participants to categorize emotion and gender of the faces. These tasks were presented in an alternate-run (Rogers and Monsell, 1995) and were counterbalanced across participants (EEGGEE/GGEEGG) whereas E represents emotion and G represents gender task. E/G is the first trial that will be excluded as there was no switch on the first trial. Half of the participants started the experiment with emotion task whereas the other half started the experiment with gender task first. The experiment was designed in E-prime software (Schneider et al., 2002) and was displayed on 16-inch laptop screen. Each task was presented with a cue as a colored background as black color was used as a cue to perform emotion categorization while blue color served a cue to indicate that the task at hand is gender categorization. Participants were instructed to categorize emotion whether the face depicted happy or a neutral expression. In gender task, they must categorize faces as male or female by pressing the fixed keys on the keyboard. For example, male = 1, female = 2, happy = 3, and neutral = 4. Errors can be task irrelevant if keys pertaining to the other task will be pressed, for example pressing key 3 or 4 during gender categorization. Errors can be recorded as category irrelevant when keys pertaining to the opposite category will be pressed such as pressing 2 when responding male face during gender categorization. Each trial started with a fixation in the middle of screen for 1000 ms, and then a gray blank screen for another 1000 ms, followed by appearance of face which remained on the screen until the response was generated.

##### Trial configuration

**Figure 1:**
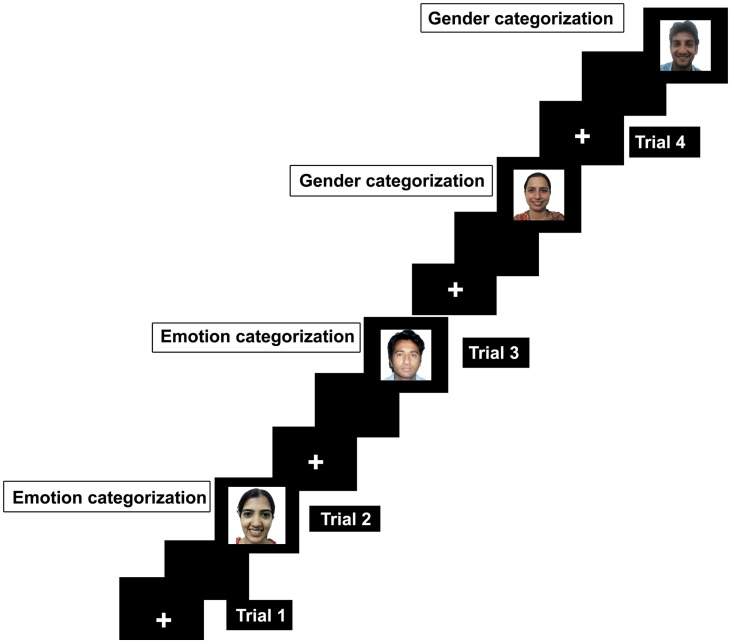
**Schematic diagram for trial sequence**.

The experiment was designed with 241 trials. Of 241 trials, the first trial was a no-switch trial. Therefore, the first trial will be exclusive of the final analysis. The remaining 240 trials had 120 switch trials (60 emotion categorization; 60 gender categorization) and 120 no-switch trials (60 emotion categorization; 60 gender categorization), Figure 1. The switch occurred in every second trial in the experiment (EE*GGE*E*G*G). The background color of the screen was changed every second trial to indicate the switched task.

#### Emotion regulation questionnaire

Emotion regulation questionnaire (ERQ) is a measure of the respondent’s tendency to regulate their emotions in stressful situations and daily life. It is a 10-item scale that can be scored on a 7-point Likert scale: from 7 (strongly agree) to 1 (strongly disagree). The subscales are cognitive reappraisal and expressive suppression. Cognitive reappraisal assesses how the respondent positively regulates emotions to reduce psychological impact of the current situation. The scale consists of six items (1,3,5,7,8,10). Emotion suppression examines how the respondent consciously hides/conceals uncomfortable feelings/thoughts in an adaptable way. The scale consists of four items (2,4,6,9). The ERQ has appropriate psychometric properties with alpha reliabilities of reappraisal 0.79 and suppression 0.73. The test–retest reliability was 0.69, with high internal consistency for cognitive reappraisal (α = 0.79) and emotion suppression (α = 0.73; Gross and John, 2003).

### Procedure

The study was approved by board of studies of the Islamia University of Bahwalpur. Informed consent was obtained from participants. Following, they were asked to complete ERQ. Subsequently, they were told about the experimental procedure. They were instructed that their speed and accuracy for face categorizations were measured and recorded. For the emotion task, they must categorize faces as happy or neutral. For gender task, faces must be categorized as male or female. They must complete the experiment quickly without compromising the response accuracy. Participants completed 241 experimental trials and were debriefed. Finally they were thanked for their participation.

### Data reduction and analyses

Analysis was conducted with the statistical package of SPSS version 20 (IBM, 2011): (i) RTs and errors were removed exceeding 2.5 standard deviations from each participant’s mean. RTs and errors for the first trial were not included in the analysis because there was no task switch on the first trial. Only RTs for correct trials were included in the analysis. The switch costs (mean RTs on switch trials subtracted from mean RTs on repeat trials) for emotion and gender tasks were calculated.

To test differences in switch cost between EBP and healthy subjects, we performed a 2 × 2 × 2 repeated measures ANOVA with trial (switch; task changes from the previous trial vs. repeat; task remains same as the previous trial) and task (emotion categorization vs. gender categorization) as within-subject factors, and group (EBP vs. healthy control subjects) as between-subject factors. (ii) Pairwise *t*-test comparisons were computed to test differences between EBP and healthy subjects on emotion regulation strategies. (iii) To test the relationship between switch costs and emotion regulation, we conducted regression analysis with emotion regulation as independent and switch costs as dependent variables.

## Results

### Task-switching data

#### Reaction times

**Figure 2:**
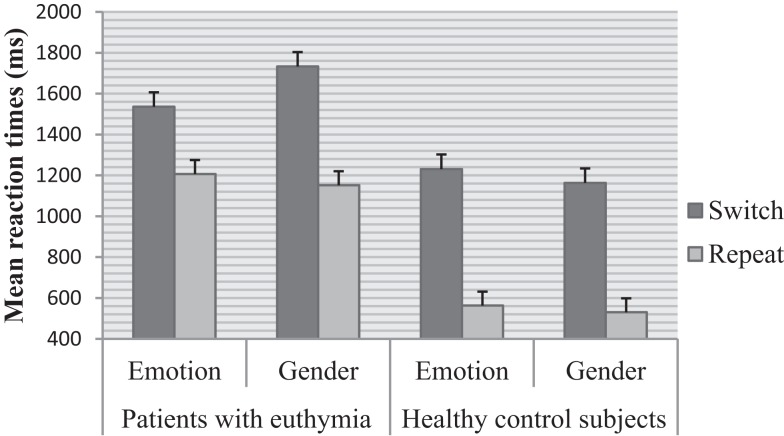
**Mean reaction times for EBP and controls**. Error bars represent standard errors.

Main effects of trial *F* (1, 78) = 562.00, *p* < 0.001, ηp2 = 0.87 and group *F* (1, 78) = 30.28, *p* < 0.001, ηp2 = 0.28 were significant. Switch trials were performed slower than repeat trials. EBP performed slower than healthy control subjects. The effect of task was not significant *F* (1, 78) = 0.44, *p* = 0.50, ηp2 = 0.00 (see Table 2 for means). There was a significant higher order interaction between trial and task and group *F* (1, 78) = 44.43, *p* < 0.001, ηp2 = 0.36, Figure 2. This interaction was further analyzed through separate repeated measure ANOVAs for each group with trial (switch vs. repeat) and task (emotion vs. gender) as within- subject factors. For EBP, main effects of the trial *F* (1, 39) = 145.55, *p* < 0.001, ηp2 = 0.78 and task *F* (1, 39) = 6.65, *p* < 0.01, ηp2 = 0.14 were significant (see Table 2 for means). The interaction between trial and task was also significant *F* (1, 39) = 55.80, *p* < 0.001, ηp2 = 0.58. The switch cost for the gender task was larger than the emotion task *t* (39) = 7.47, *p* < 0.001, emotion (*M* = 328.66, SD = 258.38) and gender (*M* = 581.33, SD = 264.38). For healthy control subjects, the main effect of trial *F* (1, 39) = 561.40, *p* < 0.001, ηp2 = 0.93 and task *F* (1, 39) = 13.80, *p* < 0.001, ηp2 = 0.26 was significant, Table 2. The interaction between trial and task was not significant *F* (1, 39) = 1.81, *p* = 0.18, ηp2 = 0.04.

**Table 2:** **Mean (*M*) and standard deviations (SD) of reaction. times (ms) in task-switching experiment**.

	Total sample (*N* = 80) *M* (SD)	Patients (*n* = 40) *M* (SD)	Healthy controls (*n* = 40) *M* (SD)
Switch	1416.40 (52.71)	1635.17 (74.54)	1197.64 (75.67)
Repeat	863.56 (43.34)	1180.17 (64.12)	547.00 (65.10)
Patients	1408.00 (66.63)	–	–
Controls	872.30 (66.62)	–	–
Emotion	1135.00 (47.20)	1372.24 (66.75)	897.49 (67.42)
Gender	1145.10 (48.15)	1443.10 (68.10)	847.10 (70.17)

#### Errors

Errors are presented in Table 3. Errors occurred when either task or category irrelevant manual keys were pressed.

**Table 3:** **Mean error rates (*M*) and standard deviations (SD) in task-switching experiment**.

Group	Emotion task		Gender task	
	Switch *M* (SD)	Repeat *M* (SD)	Switch *M* (SD)	Repeat *M* (SD)
**TASK IRRELEVANT**				
Patients	0.09 (0.05)	0.07 (0.02)	0.09 (0.05)	0.15 (0.01)
Control	0.05 (0.01)	0.03 (0.01)	0.17 (0.01)	0.03 (0.01)
**CATEGORY IRRELEVANT**				
Patients	0.10 (0.05)	0.04 (0.02)	0.08 (0.05)	0.02 (0.02)
Control	0.01 (0.01)	0.02 (0.05)	0.02 (0.05)	0.01 (0.05)

### Task switch costs and emotion regulation strategies

Pairwise comparisons depicted that EBP (*M* = 19.55, SD = 12.43) scored lower on cognitive reappraisal than healthy control subjects (*M* = 27.15, SD = 6.75); *t* (39) = 3.54, *p* < 0.001 whereas they scored higher on emotion suppression (*M* = 19.00, SD = 5.49) than healthy control subjects (*M* = 15.90, SD = 3.84); *t* (39) = 2.69, *p* < 0.01. A regression analysis was conducted with switch costs (i.e., difference between RTs on switch and repeat trials) as dependent variable and emotion suppression, cognitive reappraisal, group, gender, intellectual function, disease duration, frequency per week, and age as independent variables. The analysis achieved a significant result *F* (8, 79) = 7.78, *p* < 0.001, *R*^2^ = 0.46. Standard regression coefficients showed that none of the variables proved to be significant predictor except cognitive reappraisal β = −0.45, *t* = 3.91, *p* < 0.001; emotion suppression β = 0.22, *t* = 1.84, *p* = 0.06; group β = 0.01, *t* = 0.03, *p* = 0.97; gender β = 0.01, *t* = 0.12, *p* = 0.90; intellectual function β = 0.06, *t* = 0.67, *p* = 0.50; disease duration β = 0.06, *t* = 0.31, *p* = 0.75; frequency per week β = 0.90, *t* = 1.54, *p* = 0.12; and age β = 0.05, *t* = 0.12, *p* = 0.90.

## Discussion

The present study was designed to compare task-switching abilities between EBP and healthy individuals. Second, we aimed to assess the relationship between emotion regulation strategies and task-switching abilities. The study showed important results: (i) EBP showed asymmetries between emotion and gender categorizations. There was a larger switch cost for the gender than the emotion categorization. On contrary, asymmetric effects did not arise in healthy individuals. This result supported our first hypothesis. (ii) EBP showed deficits in emotion regulation. In contrast to healthy individuals, EBP reported frequent use of emotion suppression and sporadic use of cognitive reappraisal. The second hypothesis of the study was supported by these findings. (iii) Emotion regulation successfully predicted switch costs. This result reinforced the third hypothesis of the study.

Euthymia is associated with difficulties in emotion regulation (Harmer et al., 2002a) and a large body of research has focused how cognition and emotion regulation is disrupted in euthymia (e.g., Cabeza and Nyberg, 2000). However, it remains unclear how emotion regulation strategies mark task-switching abilities. The present research represents three important areas of strength. First, this study is one of the first to use a rigorous experimental approach employing task-switching paradigm. Second, we systematically measured the difficulty in switching with respect to emotion and gender categorization (non-emotion) task. Third, we examined which emotion regulation strategy can successfully explain the task-switching performance.

Euthymic bipolar patients showed asymmetric switch costs whereas such asymmetries were absent in healthy individuals. EBP showed higher error rate than healthy individuals for both tasks. EBP displayed a larger switch cost for gender than the emotion task. This result depicted task switching deficits in euthymic patients in terms of difficulty in switching attention away from the emotion task. As a result, gender categorization suffered and yielded a larger cost in RTs. Previous studies have shown that EBP perform worse on cognitive tasks that involve executive functions, memory, attention, planning (Quraishi and Frangou, 2002; Langenecker et al., 2010; Pattanayak et al., 2012; Camelo et al., 2013), and response inhibition (Townsend et al., 2007). EBP are deficient in cognitive flexibility and set-shifting (Ozdel et al., 2007; Ryan et al., 2012; Miguélez-Pan et al., 2014). The differential activations in vlPFC are associated with assimilation of emotion-related information (Cabeza and Nyberg, 2000). The dysfunction in this brain area results in emotion dysregulation among euthymic patients (Harmer et al., 2002b). Neuropsychological data demonstrate hyperactivation in cortico-limbic pathways (Cerullo et al., 2009) and a loss of white matter connectivity between fronto-limbic areas specifically involving prefrontal and frontal regions (Regenold et al., 2007; Brambilla et al., 2009; Heng et al., 2010). Reduced connections between cortico-limbic areas suggest that EBP have deficits in cognitive control specifically when an emotion task is involved. Our results have shown that the attentional system in EBP is biased toward emotion-related information, therefore the emotion task captured greater attention than the non-emotion task. As a result, the non-emotion task had larger switch cost. This finding is in line with previous studies that showed reduced activity in cortical regions and vlPFC during the performance of an emotion labeling task (Foland-Ross et al., 2012). A differential pattern of asymmetric switch costs can be seen in the context of differential neural activity between euthymic patients and healthy individuals in response to emotional faces (Hassel et al., 2008). EBP have diminished activity in vlPFC during response inhibition (Townsend et al., 2007; Kaladjian et al., 2009).

Our results showed preferential attention for emotion categorization among euthymic patients, which delayed gender categorizations. These findings are consistent with previous studies, which illustrated that positive mood impairs switching abilities due to disinhibition of task irrelevant information in the working memory (Phillips et al., 2002). As a result, greater flexibility for processing task irrelevant information is seen (Dreisbach and Goschke, 2004; Goeleven et al., 2007; Rowe et al., 2007). Our finding also asserts that EBP lacks in inhibition of emotion task when there is a need to switch to another task. This finding is consistent with studies that have shown greater cognitive flexibility and lack of inhibitory response in positive affect (Isen and Daubman, 1984; Isen et al., 1987, 1992; Greene and Noice, 1988). The lesser switch cost for emotion than the gender task shows that emotion task is probably more motivational for EBP. As Savine and Braver (2010) demonstrated that performance on incentive trials is associated with activations of reward centers of the brain (i.e., dopaminergic midbrain, paracingulate cortex) that results in low switch costs.

Euthymic bipolar patients suffer from difficulties in regulation and subjective experience of emotions (Lezak, 2004). Our results showed that euthymic patients use different emotion regulation strategies than healthy control subjects. In contrast with healthy control subjects, euthymic patients reported frequent use of emotion suppression while lesser use of cognitive reappraisal. Among emotion regulation strategies, cognitive reappraisal is significant predictor of task-switching abilities. Reappraisal helps in reformulation of the situation by changing the emotional response. This strategy intervenes early in the emotion-generative process and recruits brain areas involved in the executive cognitive control such as medial, dorsolateral, ventrolateral PFC, and dorsal anterior cingulate cortex (Ochsner et al., 2002, 2004). As switching in this study required the downregulation of emotion in order to switch to the non-emotion task, reappraisal successfully predicted the switch costs. Reappraisal downregulates the emotion-related experience and decreases amygdala response. In contrast, emotion suppression increases amygdala response and occurs late in PFC (Ochsner and Gross, 2005; Goldin et al., 2008).The regular use of reappraisal leads to enhanced cognitive control of emotion, psychological, and physiological well-being (Gross and John, 2003).

### Limitations and future directions

The present study is first to highlight switching deficits in euthymic patients. Results of the present study are of significant importance for future research in the following context: (1) the cognitive control of facial attributes can be further explored, for example, how face perception affects social interaction in euthymic patients, (2) whether switching abilities among euthymic patients can be improved with training, and (3) understanding neural bases of task switching may help clinical practice to formulate psychotherapies for euthymic patients. However, here we used emotion categorization as positive and neutral expressions and it is unknown whether results will generalize to other types of emotions and emotional stimuli. One important direction for future research clearly is to consider switching to and from affective states in euthymic individuals. In the present study, we focused emotion suppression and cognitive reappraisal as emotion regulation strategies. This could be a constraint on our conclusions. Future research must consider other emotion regulation patterns in EBP for wider generalization of results.

## Conflict of Interest Statement

The authors declare that the research was conducted in the absence of any commercial or financial relationships that could be construed as a potential conflict of interest.

## References

[B1] Alvarez J. A. Emory E. (2006). Executive function and the frontal lobes: a meta-analytic review. Neuropsychol. Rev. 16, 17–42. 10.1007/s11065-006-9002-x 16794878

[B2] American Psychiatric Association. (2013). Diagnostic and Statistical Manual of Mental Disorders American Psychiatric Association, 5th Edn. Arlington: American Psychiatric Publishing, 123–154

[B3] Anderson I. M. Haddad P. M. Scott J. (2012). Bipolar disorder. BMJ 345, e8508. 10.1136/bmj.e8508 23271744

[B4] Aron A. R. Monsell S. Sahakian B. J. Robbins T. W. (2004). A componential analysis of task-switching deficits associated with lesions of left and right frontal cortex. Brain 127, 1561–1573. 10.1093/brain/awh169 15090477

[B5] Becerra R. Cruise K. Murray G. Bassett D. Harms C. Allan A. (2013). Emotion regulation in bipolar disorder: are emotion regulation abilities less compromised in euthymic bipolar disorder than unipolar depression or anxiety disorders? Open J. Psychiatry 3, 1–7. 10.4236/ojpsych.2013.34A001

[B6] Brambilla P. Bellani M. Soares J. C. Tansella M. (2009). White matter connectivity in bipolar disorder. Int. Rev. Psychiatry 21, 380–386. 10.1080/09540260902962172 20374151

[B7] Brass M. von Cramon D. Y. (2002). Fractionating the neural substrate of cognitive control processes. Proc. Natl. Acad. Sci. U.S.A. 99, 14595–14600. 10.1073/pnas.222193299 12391312 PMC137928

[B8] Butler E. A. Egloff B. Wilhelm F. H. Smith N. C. Erickson E. A. Gross J. J. (2003). The social consequences of expressive suppression. Emotion 3, 48–67. 10.1037/1528-3542.3.1.48 12899316

[B9] Cabeza R. Nyberg L. (2000). Imaging cognition II: an empirical review of 275 PET and fMRI studies. J. Cogn. Neurosci. 12, 1–47. 10769304 10.1162/08989290051137585

[B10] Camelo E. V. M. Velasques B. Ribeiro P. Netto T. Cheniaux E. (2013). Attention impairment in bipolar disorder: a systematic review. Psychol. Neurosci 6, 10.3922/j.psns.2013.3.08

[B11] Cerullo M. A. Adler C. M. Delbello M. P. Strakowski S. M. (2009). The functional neuroantomy of the bipolar disorder. Int. Rev. Psychiatry 21, 314–322. 10.1080/09540260902962107 20374146

[B12] Chu O. (2014). “The effect of mood on set-switching abilities in younger and older adults,” in Electronic Theses and PHD Dissertation, (Windsor, ON: University of Windsor). Paper 5013, 29–38

[B13] Dalgleish T. (2004). The emotional brain. Nat. Rev. Neurosci. 5, 582–589. 10.1038/nrn1432 15208700

[B14] Dreisbach G. Goschke T. (2004). How positive affect modulates cognitive control: reduced perseveration at the cost of increased distractibility. J. Exp. Psychol. Learn. Mem. Cogn. 30, 343–353. 10.1037/0278-7393.30.2.343 14979809

[B15] Ellis H. C. Ashbrook P. W. (1988). “Resource allocation model of the effects of depressed mood states on memory,” in Affect, Cognition and Social Behavior, eds Fiedler K. Forgas J. F. (Gottinen: Hogrefe), 25–48

[B16] Feldman G. C. Joormann J. Johnson S. L. (2008). Responses to positive affect: a self-report measure of rumination and dampening. Cognit. Ther. Res. 32, 507–525. 10.1007/s10608-006-9083-0 20360998 PMC2847784

[B17] Fiedler K. (2001). “Affective influences on social information processing,” in Handbook of Affect and Social Cognition, ed. Forgas J. P. (Mahwah, NJ: Lawrence Erlbaum Associates Publishers), 163–185

[B18] Foland-Ross L. C. Bookheimer S. Y. Liberman M. D. Sugar C. A. Townsend J. Fischer J. (2012). Normal amygdala activation but deficient ventrolateral prefrontal activation in adults with bipolar disorder during euthymia. Neuroimage 59, 738–744. 10.1016/j.neuroimage.2011.07.054 21854858 PMC3216485

[B19] Forbes E. E. Miller A. Cohn J. F. Fox N. A. Kovacs M. (2005). Affect-modulated startle in adults with childhood-onset depression: relations to bipolar course and number of lifetime depressive episodes. Psychiatry Res. 134, 11–25. 10.1016/j.psychres.2005.01.001 15808286

[B20] Gazzaley A. D’Esposito M. (2007). “Unifying prefrontal cortex function: executive control, neural networks, and top-down modulation,” in The Human Frontal Lobes: Functions and Disorders, 2nd Edn. eds Miller B. L. Cummings J. L. (New York, NY: Guilford Press), 187–206

[B21] Goeleven E. De Raedt R. Koster E. H. W. (2007). The influence of induced mood on the inhibition of emotional information. Motiv. Emot. 31, 208–218. 10.1007/s11031-007-9064-y

[B22] Goldin P. R. McRae K. Ramel W. Gross J.J. (2008). The neural bases of emotion regulation: reappraisal and suppression of negative emotion. Biol. Psychiatry 631, 577–586. 10.1016/j.biopsych.2007.05.031 17888411 PMC2483789

[B23] Goschke T. (2003). “Voluntary action and cognitive control from a cognitive neuroscience perspective,” in Voluntary Action. An Issue at the Interface of Nature and Culture, eds Maasen S. Prinz W. Roth G. (Oxford: Oxford University Press), 49–85

[B24] Greenberg J. Meiran N. (2014). The role of emotional engagement and mood valence in retrieval fluency of mood incongruent autobiographical memory. Front. Psychol. 5:83. 10.3389/fpsyg.2014.00083 24570671 PMC3916766

[B25] Greene T. R. Noice H. (1988). Influence of positive affect upon creative thinking and problem solving in children. Psychol. Rep. 63, 895–898. 10.1111/j.2044-8279.2011.02052.x 23369173

[B26] Gross J. J. John O. P. (2003). Individual differences in two emotion regulation processes: implications for affect, relationships, and well-being. J. Pers. Soc. Psychol. 85, 348–362. 10.1037/0022-3514.85.2.348 12916575

[B27] Gross J. J. Levenson R. W. (1993). Emotional suppression: physiology, self -report, and expressive behavior. J. Pers. Soc. Psychol. 64, 970–986. 10.1037/0022-3514.64.6.970 8326473

[B28] Gross J. J. Munoz R. F. (2006). Emotion regulation and mental health. Clin. Psychol. Sci.Pract. 2, 151–164. 10.1111/j.1468-2850.1995.tb00036.x

[B29] Gruber J. Johnson S. L. Oveis C. Keltner D. (2008). Risk for mania and positive emotional responding: too much of a good thing? Emotion 8, 23–33. 10.1037/1528-3542.8.1.23 18266513 PMC2847501

[B30] Gyurak A. Goodkind M. S. Kramer J. H. Miller B. L. Levenson R. W. (2012). Executive functions and the up-regulation and down-regulation of emotion. Cogn. Emot. 26, 103–118. 10.1080/02699931.2011.557291 21432634 PMC3155745

[B31] Hamilton M. A. (1960). Rating scale for depression. J. Neurol. Neurosurg. Psychiatr. 12, 56–62. 10.1136/jnnp.23.1.56 14399272 PMC495331

[B32] Harmer C. J. Clark L. Grayson L. Goodwin G. M. (2002a). Sustained attention deficit in bipolar disorder is not a working memory impairment in disguise. Neuropsychologia 40, 1586–1590. 10.1016/S0028-3932(02)00019-2 11985840

[B33] Harmer C. Grayson L. Goodwin G. (2002b). Enhanced recognition of disgust in bipolar illness. Biol. Psychiatry 51, 1091–1099. 10.1016/S0006-3223(01)01249-5 11958780

[B34] Harris C. R. (2001). Cardiovascular responses of embarrassment and effects of emotional suppression in a social setting. J. Pers. Soc. Psychol. 81, 886–897. 10.1037/0022-3514.81.5.886 11708564

[B35] Hassel S. Almeida J. R. Kerr N. Nau S. Ladouceur C. D. Fissell K. (2008). Elevated striatal and decreased dorsolateral prefrontal cortical activity in response to emotional stimuli in euthymic bipolar disorder: no associations with psychotropic medication load. Bipolar Disord. 10, 916–927. 10.1111/j.1399-5618.2008.00641.x 19594507 PMC2711546

[B36] Heng S. Song A. W. Sim K. (2010). White matter abnormalities in bipolar disorder: insights from diffusion tensor imaging studies. J. Neural Transm. 117, 639–654. 10.1007/s00702-010-0368-9 20107844

[B37] Hollon S. D. DeRubeis R. J. Seligman M. E. P. (1992). Cognitive therapy and the prevention of depression. Appl. Prev. Psychol. Curr. Sci. Perspect. 1, 89–96. 10.1016/S0962-1849(05)80149-0

[B38] IBM. (2011). IBM SPSS Statistics for Windows, Version 20.0. Armonk, NY: IBM Corp

[B39] Isen A. M. Daubman K. A. (1984). The influence of affect on categorization. J. Pers. Soc. Psychol. 47, 1206–1217. 10.1037/0022-3514.47.6.1206

[B40] Isen A. M. Daubman K. A. Nowicki G. P. (1987). Positive affect facilitates creative problem solving. J. Pers. Soc. Psychol. 52, 1122–1131. 10.1037/0022-3514.52.6.1122 3598858

[B41] Isen A. M. Niedenthal P. Cantor N. (1992). The influence of positive affect on social categorization. Motiv. Emot. 16, 65–78. 10.1007/BF00996487

[B42] Jersild A. T. (1927). Mental set and shift. Arch. Psychol. 89, 5–82

[B43] Jhonson D. R. (2009). Emotional attention set-shifting and its relationship to anxiety and emotion regulation. Emotion 9, 681–690. 10.1037/a0017095 19803590

[B44] Johnson S. L. McKenzie G. McMurrich S. (2008). Ruminative responses to positive and negative affect among students diagnosed with bipolar disorder and major depressive disorder. Cognit. Ther. Res. 32, 702–713. 10.1007/s10608-007-9158-6 20360996 PMC2847777

[B45] Kaladjian A. Jeanningros R. Azorin J. M. Nazarian B. Roth M. Mazzola-Pomietto P. (2009). Reduced brain activation in euthymic bipolar patients during response inhibition: an event-related fMRI study. Psychiatry Res. 173, 45–51. 10.1016/j.pscychresns.2008.08.003 19442494

[B46] Langenecker S. A. Saunders E. F. Kade A. M. Ransom M. T. McInnis M. G. (2010). Intermediate: cognitive phenotypes in bipolar disorder. J. Affect. Disord. 122, 285–293. 10.1016/j.jad.2009.08.018 19800130 PMC3773480

[B47] Lezak M. (2004). Neuropsychological Assessment. New York: Oxford University Press

[B48] Magen E. Gross J. J. (2007). Harnessing the need for immediate gratification: cognitive reconstrual modulates the reward value of temptations. Emotion 7, 415–428. 10.1037/1528-3542.7.2.415 17516818

[B49] Martínez-Arán A. Vieta E. Reinares M. Colom F. Torrent C. Sánchez-Moreno J. (2004). Cognitive function across manic or hypomanic, depressed, and euthymic states in bipolar disorder. Am. J. Psychiatry 161, 262–270. 10.1176/appi.ajp.161.2.262 14754775

[B50] Mayr U. Kliegl R. (2000). Task-set switching and long-term memory retrieval. J. Exp. Psychol. Learn. Mem. Cogn. 26, 1124–1140. 11009248 10.1037//0278-7393.26.5.1124

[B51] McRae K. Jacobs S. E. Ray R. D. John O. P. Gross J. J. (2012). Individual differences in reappraisal ability: links to reappraisal frequency, well-being, and cognitive control. J. Res. Pers. 46, 2–7. 10.1016/j.jrp.2011.10.003

[B52] Miguélez-Pan M. Pousa E. Cobo J. Duño R. (2014). Cognitive executive performance influences functional outcome in euthymic type I bipolar disorder outpatients. Psichothema. 26, 166–173. 10.7334/psicothema2013.111 24755016

[B53] Monchi O. Petrides M. Doyon J. Postuma R. B. Worsley K. Dagher A. (2004). Neural bases of set-shifting deficits in Parkinson’s disease. J. Neurosci. 24, 702–710. 10.1523/JNEUROSCI.4860-03.2004 14736856 PMC6729250

[B54] Murphy F. C. Rubinsztein J. S. Michael A. Rogers R. D. Robbins T. W. Paykel E. S. (2001). Decision-making cognition in mania and depression. Psychol. Med. 31, 679–693. 10.1017/S0033291701003804 11352370

[B55] Murphy F. C. Sahakian B. J. Rubinsztein J. S. Michael A. Rogers R. D. Robbins T. W. (1999). Emotional bias and inhibitory control processes in mania and depression. Psychol. Med. 29, 1307–1321. 10.1017/S0033291799001233 10616937

[B56] Ochsner K. N. Bunge S. A. Gross J. J. Gabrieli J. D. (2002). Rethinking feelings: an fMRI study of the cognitive regulation of emotion. J. Cogn. Neurosci. 14, 1215–1229. 10.1162/089892902760807212 12495527

[B57] Ochsner K. N. Gross J. J. (2005). The cognitive control of emotion. Trends Cogn. Sci. 9, 242–249. 10.1016/j.tics.2005.03.010 15866151

[B58] Ochsner K. N. Ray R. D. Cooper J. C. Robertson E. R. Chopra S. Gabrieli J. D. (2004). For better or for worse: neural systems supporting the cognitive down- and up-regulation of negative emotion. Neuroimage 23, 483–499. 10.1016/j.neuroimage.2004.06.030 15488398

[B59] Ozdel O. Karadag F. Atesci F. C. Oguzhanoglu N. K. Cabuk T. (2007). Cognitive functions in euthymic patients with bipolar disorder. Ann. Saudi Med. 27, 273–278. 10.4103/0256-4947.51487 17684432 PMC6074289

[B60] Pattanayak R. D. Sagar R. Mehta M. (2012). Neuropsychological performance in euthymic Indian patients with bipolar disorder type I: correlation between quality of life and global functioning. Psychiatry Clin. Neurosci. 66, 553–563. 10.1111/j.1440-1819.2012.02400.x 23252921

[B61] Peake P. K. Hebl M. Mischel W. (2002). Strategic attention deployment for delay of gratification in working and waiting situations. Dev. Psychol. 38, 313–326. 10.1037/0012-1649.38.2.313 11881765

[B62] Phillips L. H. Bull R. Adams E. Fraser L. (2002). Positive mood and executive function: evidence from stroop and fluency tasks. Emotion 2, 12–22. 10.1037/1528-3542.2.1.12 12899364

[B63] Phillips M. L. Ladouceur C. D. Drevets W. C. (2008). A neural model of voluntary and automatic emotion regulation: implications for understanding the pathophysiology and neurodevelopment of bipolar disorder. Mol. Psychiatry 13, 833–887. 10.1038/mp.2008.65 18574483 PMC2745893

[B64] Pyszczynski T. Greenberg J. (1987). Self-regulatory perseveration and the depressive self-focusing style: a self-awareness theory of reactive depression. Psychol. Bull. 102, 122–138. 10.1037/0033-2909.102.1.122 3615702

[B65] Quraishi S. Frangou S. (2002). Neuropsychology of bipolar disorder: a review. J. Affect. Disord. 72, 209–226. 10.1016/S0165-0327(02)00091-5 12450638

[B66] Ravens J. C. (1938). Guide to Using Progressive Matrices. London: The Director of Psychological Research

[B67] Regenold W. T. Phatak P. Marano C. M. Gearhart L. Viens C. H. Hisley K. C. (2007). Myelin staining of deep white matter in the dorsolateral prefrontal cortex in schizophrenia, bipolar disorder, and unipolar major depression. Psychiatry Res. 151, 179–188. 10.1016/j.psychres.2006.12.019 17433451

[B68] Richards J. M. Butler E. A. Gross J. J. (2003). Emotion regulation in romantic relationships: the cognitive consequences of concealing feelings. J. Soc. Pers. Relation. 20, 599–620. 10.1177/02654075030205002

[B69] Rogers R. D. Monsell S. (1995). Costs of a predictable switch between simple cognitive tasks. J. Exp. Psychol. 124, 207–231. 10.1037/0096-3445.124.2.207

[B70] Rowe G. Hirsh J. B. Anderson A. K. Smith E. E. (2007). Positive affect increases the breadth of attentional selection. Proc. Natl. Acad. Sci. U.S.A. 104, 383–388. 10.1073/pnas.0605198104 17182749 PMC1765470

[B71] Royall D. R. Lauterbach E. C. Cummings J. L. Reeve A. Rummans T. A. Kaufer D. I. (2002). Executive control function: a review of its promise and challenges for clinical research. A report from the Committee on Research of the American Neuropsychiatric Association. J. Neuropsychiatry Clin. Neurosci. 14, 377–405. 10.1176/appi.neuropsych.14.4.377 12426407

[B72] Rubinstein J. S. Meyer D. E. Evans J. E. (2001). Executive control of cognitive processes in task switching. J. Exp. Psychol. Hum. Percept. Perform. 27, 763–797. 10.1037//0096-1523.27.4.763 11518143

[B73] Ryan K. A. Vederman A. C. McFadden E. M. Weldon A. L. Kamali M. Langenecker S. A. (2012). Differential executive functioning performance by phase of bipolar disorder. Bipolar Disord. 14, 527–536. 10.1111/j.1399-5618.2012.01032.x 22834461 PMC3773478

[B74] Savine A. C. Braver T. S. (2010). Motivated cognitive control: reward incentives modulate preparatory neural activity during task-switching. J. Neurosci. 30, 10294–10305. 10.1523/JNEUROSCI.2052-10.2010 20685974 PMC2935640

[B75] Sawada Y. Nishio Y. Suzuki K. Hirayama K. Takeda A. Hosokai Y. (2012). Attentional set-shifting deficit in Parkinson’s disease is associated with prefrontal dysfunction: an FDG-PET study. PLoS ONE 7:e38498. 10.1371/journal.pone.0038498 22685575 PMC3369918

[B76] Schmeichel B. J. Demaree H. A. (2010). Working memory capacity and spontaneous emotion regulation: high capacity predicts self-enhancement in response to negative feedback. Emotion 10, 739–744. 10.1037/a0019355 21038959

[B77] Schmeichel B. J. Volokhov R. Demaree H. A. (2008). Working memory capacity and the self-regulation of emotional expression and experience. J. Pers. Soc. Psychol. 95, 1526–1540. 10.1037/a0013345 19025300

[B78] Schneider D. W. Logan G. D. (2005). Modeling task switching without switching tasks: a short-term priming account of explicitly cued performance. J. Exp. Psychol. 134, 343–367. 10.1037/0096-3445.134.3.343 16131268

[B79] Schneider W. Eschman A. Zuccolotto A. (2002). E-prime User’s Guide. Pittsburgh, PA: Psychology software tools, Inc.

[B80] Stawski R. S. Almeida D. M. Lachman M. E. Tun P. A. Rosnick C. B. (2010). Fluid cognitive ability is associated with greater exposure and smaller reactions to daily stressors. Psychol. Aging 25, 330–342. 10.1037/a0018246 20545418 PMC2896229

[B81] Stuss D. T. Levine B. (2002). Adult clinical neuropsychology: lessons from studies of the frontal lobes. Annu. Rev. Psychol. 53, 401–433. 10.1146/annurev.psych.53.100901.135220 11752491

[B82] Thompson J. M. Gallagher P. Hughes J. H. Watson S. Gray J. M. Young A. H. (2005). Neurocognitive impairment in euthymic patients with bipolar affective disorder. Br J. Psychiatry 186, 32–40. 10.1192/bjp.186.1.32 15630121

[B83] Townsend J. Altshuler L. Cohen M. Eisenberger N. Foland L. Bookheimer S. (2007). “Persistent deficits in orbitofrontal cortex function in euthymic bipolar subjects,” in Conference of Society for Neuroscience 37th Annual Meeting. San Diego

[B84] von Hippel W. Gonsalkorale K. (2005). “That is bloody revolting!” Inhibitory control of thoughts better left unsaid. Psychol. Sci. 16, 497–500. 10.1111/j.0956-7976.2005.01563.x 16008778

[B85] Whitmer A. J. Gotlib I. H. (2012). Switching and backward inhibition in major depressive disorder: the role of rumination. J. Abnorm. Psychol. 121, 570–578. 10.1037/a0027474 22468767 PMC11877650

[B86] Yamasaki H. LaBar K. S. McCarthy G. (2002). Dissociable prefrontal brain systems for attention and emotion. Proc. Natl. Acad. Sci. U.S.A. 99, 11447–11451. 10.1073/pnas.182176499 12177452 PMC123276

[B87] Young R. Biggs J. Ziegler V. Meyer D. (1978). A rating scale for mania: reliability, validity and sensitivity. Br. J. Psychiatry 133, 429–435. 10.1192/bjp.133.5.429 728692

